# Design of Secure ECG-Based Biometric Authentication in Body Area Sensor Networks

**DOI:** 10.3390/s16040570

**Published:** 2016-04-22

**Authors:** Steffen Peter, Bhanu Pratap Reddy, Farshad Momtaz, Tony Givargis

**Affiliations:** Center for Embedded and Cyber-Physical Systems, University of California, Irvine, CA 92697-3455, USA; bpreddy@uci.edu (B.P.R.); fdoktorm@uci.edu (F.M.); givargis@uci.edu (T.G.)

**Keywords:** body area sensor networks, biometric, authentication, design

## Abstract

Body area sensor networks (BANs) utilize wireless communicating sensor nodes attached to a human body for convenience, safety, and health applications. Physiological characteristics of the body, such as the heart rate or Electrocardiogram (ECG) signals, are promising means to simplify the setup process and to improve security of BANs. This paper describes the design and implementation steps required to realize an ECG-based authentication protocol to identify sensor nodes attached to the same human body. Therefore, the first part of the paper addresses the design of a body-area sensor system, including the hardware setup, analogue and digital signal processing, and required ECG feature detection techniques. A model-based design flow is applied, and strengths and limitations of each design step are discussed. Real-world measured data originating from the implemented sensor system are then used to set up and parametrize a novel physiological authentication protocol for BANs. The authentication protocol utilizes statistical properties of expected and detected deviations to limit the number of false positive and false negative authentication attempts. The result of the described holistic design effort is the first practical implementation of biometric authentication in BANs that reflects timing and data uncertainties in the physical and cyber parts of the system.

## 1. Introduction

Body area sensor networks (BANs) are a promising technology for convenience, safety, and health applications [1]. Examples for BANs include fitness trackers, smart glasses [2], vital tracking of emergency response teams [3], and medical implantable devices such as heart pacemakers and insulin pumps. Such medical and safety related body area network (BAN) applications call for a high level of access control and data protection [4,5,6,7]. However, the goal of good security in BANs is challenged by the capabilities of typical body area sensor nodes. For economical and practical reasons, the nodes are small and resource-constrained, providing only limited computation power and memory.

**Figure 1:**
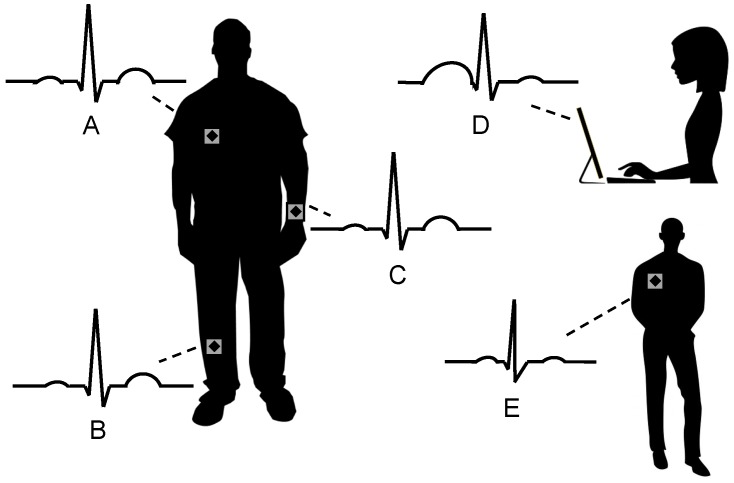
A BAN comprises sensors attached to a human body. Electrocardiography (ECG) data can ensure that sensors, attached to the same body (**A,B,C**) trust each other but do not trust sensors (**E**) and devices (**D**) that are not attached to the same body.

While security protocols and implementations exist to protect data on severely constrained devices [8,9], the question remains of how devices that belong to the same body area identify and trust each other. Figure 1 illustrates the problem: Sensors that are attached to one person (Sensor A, B, and C) should know and trust each other, while sensors attached to other persons (E) or entirely forged data (D) are not trusted. Solutions like pre-deployed keys [10] or manual setups are cumbersome and error-prone—in particular in environments with several interfering BANs.

The work in this paper addresses the challenge of identifying nodes that are physically attached to the same human body. This mechanism can be used:

- for fast and convenient setup of a BAN, for instance for fitness trackers, chest sensors, and smart wrist bands,
- for the setup of a trusted and secure body area environment with a shared key, and
- as a second authentication factor in BANs with critical implanted medical equipment, to prevent accidental or malicious erroneous access to the medical devices.

As physiological characteristic in this paper, we use ECG data. ECG records the electrical activity of the heart and is characteristic for a person at a given time. ECG and related heart data can be obtained from sensors that are attached to the body, even locally as shown in [11,12,13,14]. Literature has already discussed ECG-based authentication and key-agreement protocols [15,16,17,18], however, without considering practical implications of the low cost sensors and resource-constrained BAN platforms. Instead, existing work used clinical ECG data obtained from medical databases and processed the data on PCs, ignoring uncertainties originating from sensors and processing.

In this paper, we present a biometric authentication protocol that intrinsically reflects the statistical properties of the uncertainties, to systematically balance the risk of false rejected authentications and false accepted attempts. We address these issues in two stages:

1. Design and implementation of a sensor platform (Section 4) including suitable data processing and feature detection methods. We apply a model-based design flow [19], starting with an analytical model in Matlab, test it in real-time models on a PC (Section 5), and finally translate it to the embedded system platform, taking into account the limited resources of a BAN in Section 6.
2. Based on empirical data gathered from the implemented sensor node, we design and parametrize a secure session establishment protocol in Section 7. We show that statistical properties of the uncertainties of the system can be harnessed to improve the confidence within the authentication process.

The contribution of our work is the connection of the implementation results and the parametrization of the security protocol. We show that uncertainty in the measurements can be handled, but needs to be reflected in the security protocol to avoid a high number of false rejections or false authentications.

The result is the first biometric authentication protocol that works on actual BAN nodes. The presented system shows 100% correct authentications with a probability for a successful attack of less than 0.1%. We present the hardware design, the algorithm and software implementation, and discuss the selected security protocol parameters.

## 2. Preliminaries and Methodology

This section briefly introduces the fundamentals and terminology of ECG, and then discusses the design methodology we use throughout this paper.

### 2.1. ECG Basics

Electrocardiography (ECG) is defined as the process of recording the electrical activity of the heart over a period of time using electrodes placed on a person’s body. These electrodes detect the tiny electrical changes on the skin that arise from the heart muscle depolarizing during each heartbeat [1].

**Figure 2:**
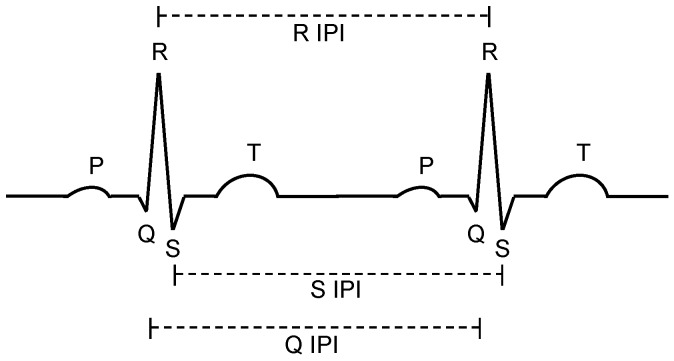
Characteristics of the heart signal and Inter-Pulse-Intervals (IPI) between peaks.

A schematic trace of two heart beats is shown in Figure 2. Dominant characteristics of the signal are the five peaks, called P, Q, R, S, and T, while the most significant peak is the R peak. All the information provided by ECG exists mainly between 0.05 Hz to 100 Hz, since the length of a Q-R-S complex is typically between 0.06 and 0.1 s [20]. While characteristics like the Q-to-R or R-to-S timing might be applied for authentication purposes, in this paper, we focus on the Inter-Pulse-Intervals (IPI). As shown in Figure 2, the IPI can be measured between two adjacent Q peaks (Q-IPI), R peaks (R-IPI) or S peaks (S-IPI). Since the typical heart-rate (HR) of a human varies between 30 and 240 beats per minute (bpm) [20], the IPI ranges between 250 ms and 2000 ms (IPI = 60 s/HR).

While the occurrence of the heart-beats is not simultaneous at each location at the body, the IPIs for a person are approximately equivalent regardless of the measured location. Therefore, the IPI is well suited for an application in BANs. The IPI is particularly interesting as it might be measured not only by ECG sensors but by optical [12], acoustic [13] or tissue pressure [14] sensors and other BAN devices.

### 2.2. Problem Statement, Methodology and Outline

The general idea of the biometric authentication protocol we introduce in Section 7 is to measure the IPIs for a certain time on different nodes. The nodes are considered to be on the same body if the measured IPIs are equivalent or very similar. The major challenges in this process are:

- uncertainty of the underlying physical (biological) phenomenon,
- timing uncertainty and jitter in the cyber-parts (sensors, interfaces, processing), and
- parametrization of the authentication protocol, to omit rejection of valid sensor pairings but reduce the probability of invalid pairing attempts to succeed due to a high tolerance of deviation.

**Figure 3:**
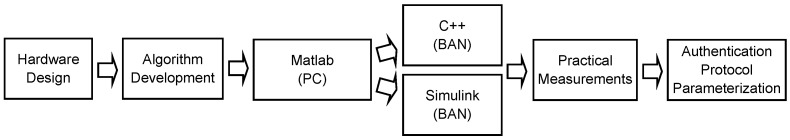
Development flow.

To address these challenges, in this paper, we pursue a model-based design flow that gravitates around real sensor data, instead of an ideal library of data. The methodology and outline of our work is shown in Figure 3. As the first step, we select hardware, including the sensors and their interfaces. The sensor access includes the design of a sensor board for analog filtering and signal pre-processing. Then, we select and implement the digital signal processing steps and the IPI detection algorithm that works with the gathered data. For first practical testing, we then use the sensor board from the Matlab implementation of the signal processing steps, running on a PC, as discussed in Section 5. For the actual BAN implementation, we apply automatic generation of the system code from the Matlab/Simulink environment, and compare it the performance to a manual implementation in C. The benefit of the Simulink approach is the seamless model-based design flow, while the main benefit of the C-implementation is its superior performance. Finally, we apply the sensor data, gathered on our prototype, to parametrize a secure biometric BAN authentication protocol. The result is a protocol that utilizes the properties of the measured sensor data to improve the confidence in the authentication.

## 3. Related Work

General security and privacy in body area networks is a complex topic that has been covered in a range of overview papers [4,5,6,21]. A sub domain of this field is the application of biometric and physiological body properties as means to establish authentication or to generate keys in such BANs. In particular ECG- and IPI- based authentication received significant research attention [15,16,17,22,23].

For instance, ECG-based signal key establishment protocol (ESKE) [16] is a noise-tolerant key generation scheme that works without pre-deployment of key material. ESKE applies wavelet filtering and requires a sample size of more than 30 s for a single authentication, which is unsuitable for most BAN scenarios. Another key agreement scheme [15] is based on the Fuzzy Vault Scheme [24]. The method allows a receiver to reconstruct a message if most coefficients of the encryption polynomial are known. A similar approach is pursued in the physiological signal based key agreement (PSKA) scheme [17], which creates a session key from the frequency information of the ECG data. However, PSKA still needs the receiver and sender to share a set of exact equivalent key values, which cannot be guaranteed in most BANs. These works are all executed on a PC and frameworks like Matlab. In addition, the methods require long sampling periods to avoid offline guessing of the key. Our work instead separates key agreement and authentication. This way we apply traditional, well established key agreement methods, such as Diffie–Hellman as a basis, and use physiological authentication as an additional step.

The ordered-physiological-feature-based key agreement (OPFKA) protocol [22] has been implemented on a BAN platform. However, OPFKA also has only been tested with medical databases, such as the MIT-BIH Arrhythmia database [25], as a source for the reference ECG signals. These works do not discuss the impact of sensing, measurement, and timing errors. Our work clearly shows the importance of coping and harnessing the measurement uncertainties. In fact, none of the presented schemes worked with the real data we gathered from our implemented sensor node.

The feasibility of IPI-based authentication for BSNs was shown in [18] and [26]. Poon [18] demonstrated the applicability of IPI-based authentication even for different types of sensors. Their experiments showed the interoperability between ECG and pulse oximeter (PPG) sensors. The work also demonstrated the suitability of the IPI authentication for older and less healthy persons. Contrary to our work, [18,26] apply binary codes that require a high number of IPI values (>30) for an authentication. They also do not consider the impact of design decisions in the signal processing and embedded systems design.

The application of physiological characteristics has also been discussed for permanent user authentication, for instance, for persistent storage in the cloud [27]. The idea is to utilize encryption keys that are based on permanent characteristics of the human physiology. While our work does not directly aim at permanent authentication, the implementation and design flow we describe can be applied to support permanent identification on BAN nodes in the future.

A range of alternative approaches have been presented to set up secure BANs and provide access and privacy protection [28]. As an example, [29] applies near field communication to ensure close proximity of BAN nodes in medical applications. Other approaches include password-authenticated key exchange and agreement [10], wristbands, proximity sensors, or using the body as a shared communication medium [30]. We consider these approaches as possible second factors in a secure authentication process for BANs.

## 4. Sensor Hardware Interface

Since the goal of this work is to use actual sensor data for the authentication, as a first step, we have to select suitable sensors, and interface the sensors to the computation nodes. One criteria at this point was not to chose clinical sensor and filter systems, which indeed are available [31] but due to cost and size are not applicable to BANs. Instead, we used low-cost sensors and designed a sensor processing board, which then can be connected to the BAN nodes.

### 4.1. Sensors

As sensors, we use conventional wet cloth electrodes with repositionable conductive adhesive hydrogel to measure the electrical activity from skin surface [32]. One main challenge of these sensors is their relatively low DC offset voltage, with raw signal amplitude below 0.5 mV. Additional noise originates internally from the device but also by the environment. As a result, initial experiments with a plain analog to digital conversion failed due to the low voltage level, a fast fading signal, and the high noise.

### 4.2. Sensor Board

**Figure 4:**
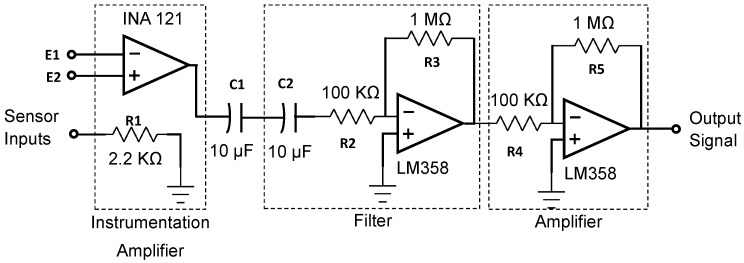
Schematic of our sensor processing board: the instrumentation amplifier obtains the difference of the sensor inputs, before the single signal is filtered and amplified.

To extract, stabilize, and clean the signal, we designed a sensor board that amplifies and filters the signals. We applied a standard difference amplifier approach [31]. The difference amplifier is a suitable solution since the basic ECG data is obtained as an output of the difference of two leads placed on the body. The block diagram of our sensor board is shown in Figure 4. The circuit consists of three parts: the differential amplifier, a filter, and a post amplifier. As a differential amplifier, we use an INA121 instrumentation amplifier, due to its high precision and high noise rejection and its sensitivity to the ECG input range. The capacitor (C1) at the output of the instrumentation amplifier stabilizes the signal by removing DC shifting. The output of the instrumentation amplifier is still noisy and contains many unwanted frequency components. Therefore, we apply an operational amplifier (LM358) to filter the ECG-characteristic frequencies. After the filter stage, signal post amplification is carried out to match the input requirements of the processing device. In other words, the range of the output signal is determined by the next device that is going to use and process the output signal. For instance, the target system we introduce in Section 6 requires input levels in the range of 1 mV to 10 mV. Based on this requirement, we use a simple inverting amplifier in which R4 and R5 are chosen appropriately for the required gain, given as G = −R5/R4. The parameters can be further adapted to reflect the input requirements of the processing platform.

### 4.3. Results

**Figure 5:**
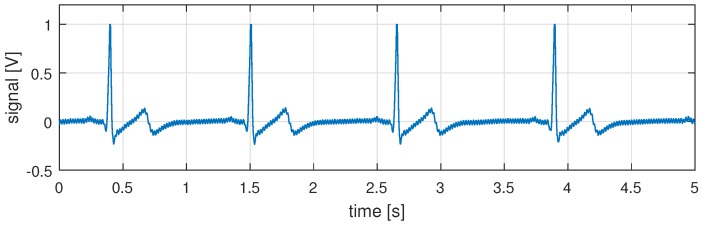
Five seconds of gathered sensor data after processing on our sensor board. Visible are the ECG characteristics, but also some residing high frequency artifacts.

A trace for data gathered with the sensor board is shown in Figure 5. The board requires 6.0 μW and has an output impedance of 1.2 MΩ. In Figure 5 we can see that the output levels are clearly distinguishable and can be processed by the subsequent embedded processing system. In the trace, the PQRST wave peaks can be identified clearly. Notable also is the steadiness of the signal level, which is important for further processing. However, we still see some high frequency components as noise. That is why, in the next step, we investigate digital signal processing and available feature detection algorithms for their suitability to work with the gathered sensor data.

## 5. Digital Signal Processing

**Figure 6:**
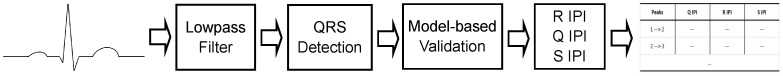
Steps of the digital processing: the ECG needs to be filtered, peaks are detected and validated. Output is a table of Q, R, and S values.

This section discusses the digital processing of the gathered ECG signal. The process is shown in Figure 6. The input is the continuous sampled ECG signal, and the output is a list of R-, Q-, and S-IPIs. To process the data, the following general steps have to be executed:

1. digital low-pass filtering to clean the ECG signal,
2. detection of the ECG features (QRS peaks), and
3. validation and correction of obtained values, based on biometric model properties.

We describe details of the three steps in the following subsections.

### 5.1. Digital Filtering

Even though the analog filters on our sensor board already filtered the signal, several high-frequency artifacts from wires, hardware and interfaces remain. As introduced in Section 2.1, all important information of the ECG signal are located between 0.05Hz to 100Hz. Such fixed cut-off frequencies can be efficiently digitally filtered using Infinite Impulse Response (IIR) butterworth filters [33]. The basic transfer function of an IIR filter is given as:

(1) $$H\left(z\right)=\frac{\sum_{i=0}^{P}b_{i}z^{-i}}{1+\sum_{j=1}^{Q}a_{j}z^{-j}}$$

while *a* and *b* are multi-dimensional characteristic coefficients of the filter, and *z* is the signal to be filtered. The generation of the coefficients for the given the cutoff frequencies and the order of the filter is well described in related work [33]. The interested reader can find the coefficients and their generation in the Matlab and C code at [34]. The actual implementation of the filter depends on the capabilities of the underlying hardware and is suspect to quality-to-resource trade-offs as we will discuss in Section 6.3.

### 5.2. ECG Feature Detection

The filtered signal is processed further to extract the features of the ECG signal. These features, which are the timing of the Q, R, and S peaks, are the core identifiers for the intended authentication scheme described in Section 7. To extract ECG features, a range of approaches have been proposed in related work [35,36]. One implementation option is the detection of the large R-peaks. However, due to the lack of redundant information, the simple R-detection leads to many unrecoverable errors and large timing uncertainties.

We apply the Pan–Tompkins real-time QRS detection algorithm (PTA) [36]. PTA extracts the QRS complex from a given ECG signal and is suitable for resource-constrained devices. PTA is also considered as robust in presence of abnormal ECGs, such as arrhytmias [37]. PTA performs a sequence of filtering and comparison steps, including:

- a five-point derivative filtering to provide the slope information of the QRS complex, using the transfer function $H\left(z\right)=\frac{1}{8}\left(-z^{-2}-2z^{-1}+2z^{1}+z^{2}\right)$,
- squaring of the signal, to obtain all positive signal values and nonlinear amplification to emphasize the characteristic higher ECG frequencies,
- fixed moving window integration to obtain waveform feature information in addition to the slope of the R wave, and
- a comparison step to identify the largest peaks in a window to locate Q, R, and S.

**Figure 7:**
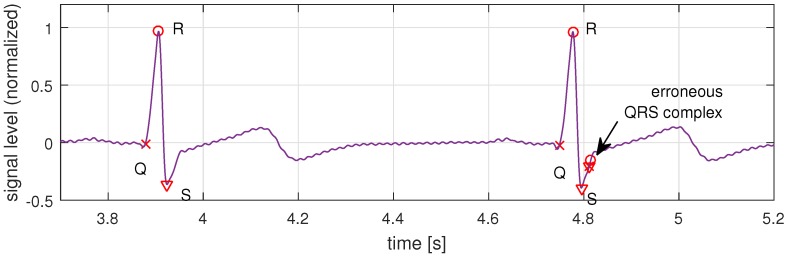
Signal and detected QRS peaks for two QRS complexes after Pan-Tompkins. Note that a third erroneous QRS complex is detected at time stamp 4.8 s.

The output of the PTA is a table of identified Q, R, and S time indices. The benefit of PTA is that each step can easily be implemented even on severely constrained embedded devices. However, one disadvantage of PTA is that the simplified computation model leads to erroneously detected peaks if applied to non-ideal input signals. One example of an erroneous QRS complex is shown after 4.8 s in Figure 7. In this example, PTA falsely identified another QRS complex just in the slope after the correct S peak. In fact, practical measurements showed errors in about 6% of our measured QRS complexes, which have to be addressed in the following model-based data validation step.

### 5.3. Model-Based Data Validation

To reduce the impact of erroneously detected peaks, we exploit data redundancy and knowledge about the typical heart beat to identify and fix these errors. The general idea is to validate that the detected QRS values are in the expected QRS order, and that the magnitudes and time differences of the detected Q, R and S peaks are within the expected theoretical range for a normalized ECG signal. Since the duration of a QRS complex is expected to be between 0.06 and 0.1 s, the distance between two consecutive Q, R, or S peaks must be greater than 0.06 s as well. These basic rules lead to Algorithm 1.

**Algorithm 1** Model-based Validation on detected QRS peaks

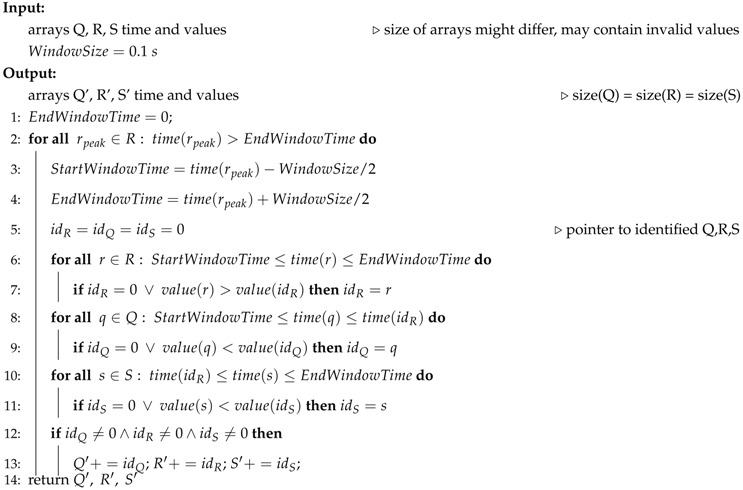



The inputs to the validation algorithm are the Q,R, and *S* locations (time, value) delivered by PTA. The algorithm iterates through the locations of detected peaks, and copies valid QRS locations into the output arrays $Q^{\prime },R^{\prime },$ and $S^{\prime }$. To identify a valid complex, a window of 0.1 s centered at R-peaks ($r_{peak}$, line 2–4) is moved over the input data. Within each window, we identify the highest R (line 6–7), lowest Q (line 8–9), and lowest S (line 10–11). Only if valid Q, R, and S locations are found with a window range, they are added to the output arrays (line 12–13), otherwise the peaks are discarded. The result is an array of valid Q, R, and S time values from which the IPI values can be computed.

Applying the presented algorithm, the false peaks in the example in Figure 7 are removed because the false R appears within the 0.1 s window after the correct R. The false R and its adjacent S then are discarded because their peaks are smaller than the correct ones, and the Q is discarded since it does not follow the expected pattern. In our tests, which we present next, the validation algorithm could fix 100% of the erroneously detected peaks.

### 5.4. Matlab Implementation

In this section, we describe the setup and the results of a first prototype system that works with the presented sensor board but performs the digital processing steps in Matlab on a PC.

#### 5.4.1. Setup

For the Matlab implementation, two steps need to be addressed: first, how to interface the sensor board, and second, how to execute the digital processing, discussed in the previous section.

To interface the sensor device with the PC, we require an analog to digital conversion that is easily accessible from Matlab. One implementation option is an external AD converter. However, for our experiments, we sampled the data via the microphone port of the PC, via a standard audio jack. The Matlab function *Audio Recorder* provides direct and convenient access to the analog input data, which performs continuous sampling between 1 kHz and 96 kHz with a precision of 8-bit to 24-bit. For our experiment, we used the settings 3 kHz and 16 bit.

Since Matlab has native support for designing filters, butterworth filters can easily be designed using in-built functions such as *butter*. The function generates the coefficients that are utilized to generate the impulse response of the filter using *impz*. With the generated filter response, we can perform the filtering on any given signal using *filtfilt*. Similarly, the steps of the Pan–Tompkins algorithm are implemented, applying the Matlab functions for convolutions and signal processing. Finally, the data validation algorithm is directly implemented as the pseudo code shown in Algorithm 1.

#### 5.4.2. Results

To assess the processing steps and the algorithms, we connected the sensor board to a PC (i5, 4GB RAM) running Matlab. The results for two sensor systems tracking the Q-, R-, and S IPIs are shown in Table 1. In Table 1, we see that both sensor systems obtain approximately the same IPIs. In addition, the Q, R, and S IPIs match each other for a given time index. The results indicate the suitability of the sensor board and the data processing steps to be applied for the intended physiological authentication of sensor nodes attached to the same person. The computation time for processing the eight IPIs in Matlab on the PC is on average 0.14 s. The memory consumption is 28 MB. To reduce the computation and memory footprint, we translate the digital processing steps from the PC to a BAN node in the next section.

## 6. Embedded System Implementation

In this section, we further follow the model-based design methodology and translate the digital ECG signal processing implementation from the PC-Matlab environment to an embedded system platform. The core challenge in this part is to cope with the limited interfaces and processing capabilities. We describe and compare the results for an automatically generated implementation from the Matlab-Simulink environment, and a manual implementation in C. Due to its superior performance, the latter one is the basis for the security protocol we describe in the next section.

### 6.1. Target Platform

As the embedded target platform in this paper, we chose a Raspberry Pi (RPi). The RPi is a low power (0.7–1.2 W) single-board computer with a 700 MHz ARM processor and 512 MB of RAM. The RPi is one of the more powerful BAN nodes, and has already been applied in a range of practical BAN and e-health applications [38,39]. One particular benefit of the RPi is it is supported by Matlab, Simulink and a range of design tools, which facilitates easy and fast prototyping.

**Figure 8:**
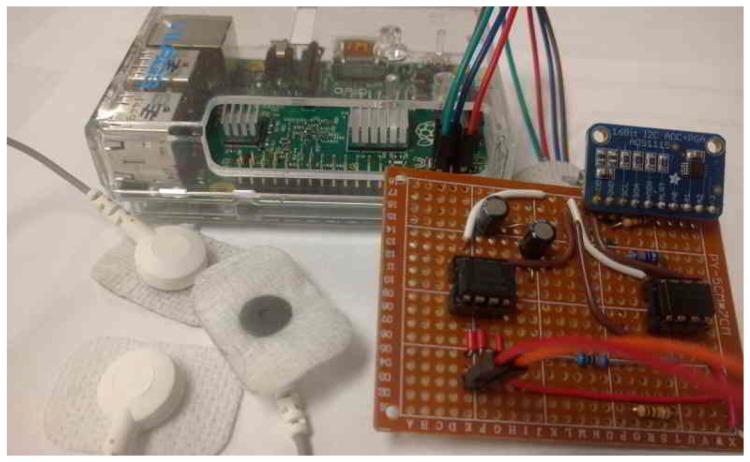
Photo of our experimental setup: electrodes with sensor board in front, the RaspberryPi board at the back.

For our prototype, we used the RPi and connected our sensor board via an external 16 bit ADC (ADS1115) to the RPi’s GPIOs, resulting in an effective measurement resolution of 9 μV, which proved to be sufficient for tracking the ECG signal. The setup of the RPi, the sensor board, and the sensors is shown in Figure 8. In the following we describe two approaches to implement the software on the system: first, automatically generated from Matlab/Simulink, and second, manually implemented in C.

### 6.2. Simulink

For the first implementation, we further followed the model-based design methodology [19], by using the Matlab system to automatically generate C-code that can be compiled for the target system. We applied the Simulink coder [40], which is an industry grade tool that generates C and C++ code from a Simulink model. The Simulink coder helps the system designer by evaluating the model and block parameters, propagating signal widths and sample times, and determination of the execution order of blocks within the model.

**Figure 9:**
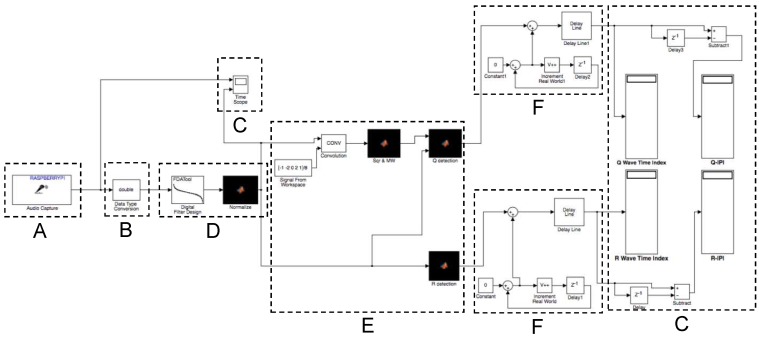
Block diagram of the Simulink Model. (**A**) data acquisition; (**B**) data conversion; (**C**) output; (**D**) lowpass filter; (**E**) Pan–Tompkins QRS detection; and (**F**) time tracker.

However, the Simulink coder still requires the manual conversion of the Matlab model to a block dataflow models in Simulink. Most parts of our system could be directly translated, since the applied filter functions and signal processing blocks are available in Simulink. The major challenge concerns the time measurement. The algorithm implemented on Simulink calculated QRS time indices only in its respective frames and not for the continuous recording over a period of time. Hence, a timer was required within the model to track the signals in real time. In the Simulink system diagram, shown in Figure 9, the timing counter is highlighted as block **F**. The other parts of the design correspond to the data processing steps discussed in the previous section and are: **A**: Data acquisition; **B**: Data conversion; **C:** Output; **D**: Lowpass filter; **E**: Pan–Tompkins QRS detection. The model is available from our project webpage [34].

For the generated code on our target platform, we obtained the following results. The Q and R IPI results of the implementation for two sensor systems are shown in Table 2. Like the Matlab implementation (Table 1), we expected similar values for all measured IPIs. However, in Table 2, we see significant variations and errors. The main reason for the IPI mismatches are delays in data acquisition and over-utilization of the computation resources. In fact, due to the limited computation resources, we had to disable the tracking of S peaks, in order to obtain at lest R and Q-IPIs. This result shows the existing limitation of model-based design. The automatically translated Simulink implementation of the processing model is not suitable for the BAN node—even on the relatively powerful RPi node. Hence, we need to translate the data processing steps into a lower level implementation language like C, manually, to address the limited resources of a BAN.

### 6.3. C-Implementation

The starting point of the manual ANSI C implementation is the generated Matlab code. Due to the use of model based design, we were able produce C code with modest effort. The model based design helped us translate the logic implemented in Matlab and Simulink to C code. Nevertheless, we had to reduce the processing overhead and memory consumption of the embedded Matlab functions with tailored lightweight C code. The two most taxing functions of the signal processing steps are filtering and data acquisition. Therefore, we discuss the filter implementation and the sampling rate trade-off in the following paragraphs.

#### 6.3.1. Filter Implementation in C

One of the most utilized functions in the data process flow is Matlab’s filter function (*filtfilt*). *filtfilt* is used for digital filtering of the input data and for various steps of the PTA. The function utilizes the defined filter characteristic given by the impulse response to shape the data. However, to achieve the same functionality we only need a convolution (y(t)=∑h(u)x(n-u)) of the input signal *x* with the fixed impulse response *h*. The filter’s impulse response is derived from the transfer function H(z), according to Equation (1). H(z) is invariant, so that there is no need to re-generate h(n) every time. Therefore, our C implementation stores *h* and eventually reduces the amount of memory and processing power, while performing as required and not losing any essential frequency components.

#### 6.3.2. Sampling Rate

The data processing steps and the PTA require the input signal for a fixed time of several seconds. Accordingly, the samples for this time period have to be stored before the data can be processed. The memory required for storing the data is determined by the sampling rate of the signal, and it affects the total memory consumption significantly. For example, for one second of ECG signal data with a sample rate of 330, an input buffer of at least 1320 bytes is needed, if double precision data types are used. Moreover, since the input signal has to be manipulated and filtered all through the algorithm, the sampling rate directly affects the processing time and power. However, a low sampling rate might affect the processing quality negatively. In order to identify a preferable sampling rate and investigate the effect on memory consumption and the processing error, we applied the algorithm on signals with different sampling rates, ranging from 150 to 2000 samples/s. The error factor in the experiment is the squared timing error of the identified Q, R, and S peaks.

**Figure 10:**
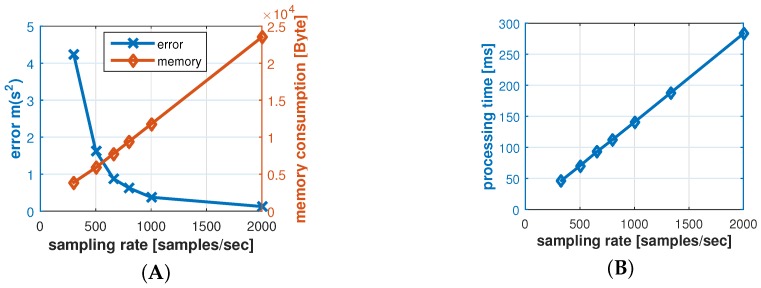
Sampling rate trade-offs: (**A**) memory to error rate; and (**B**) processing time.

The result for processing seven seconds of ECG data is shown in Figure 10A,B. The results shown in Figure 10A confirm that the error factor decreases with an increase in sampling rate, while the memory usage increases. While the memory consumption grows linearly with an increased sampling rate, the error rate decreases slower with increased rates. As a result, increasing the sampling rate beyond 500 Hz only improves the processing marginally. Hence, for our implementation, we used a sampling rate of 800 Hz and required a data memory amount of approximately 10 kB per second. As we will discuss in Section 7.4, the sampling rate of 800 Hz is preferable for the proposed authentication protocol due to its low deviation of sensor readings.

Figure 10B shows the computation efforts on our target platform for the different sampling rates for processing the data. It can be seen that the processing efforts increase about linearly with higher sampling rates, from 23 ms at 150 Hz sampling rate to 280 ms at 2000 Hz. As we will see in Section 7, the signal processing is the dominant processing consumer in our system, so that the 113 ms required for the preferred 800 Hz sampling rate is well in line with the system requirements.

#### 6.3.3. Results and Evaluation

Using the mentioned techniques, we were able to reduce the memory usage from 28 MB required for the generated Simulink implementation to 16 KB for the C-based implementation. The average CPU utilization of the RPi dropped from more than 70% required for Simulink to less than 5% for the C-based implementation.

In this section, we describe the measurement results for the C-implementation on the RPi with the sensor board (see Figure 8). For our tests, we used two sensor systems to gather the data required for the authentication process. During the tests, we validated the general functionality and quality of the measurements. The data was gathered from the four authors of this paper. The two independent sensors were attached to the left and right wrist, respectively, with separate reference signals at the chest area. For the tests, the data was synchronized by time stamps, stored in text files that could be analyzed offline. In total, we gathered 800 IPI samples.

**Figure 11:**
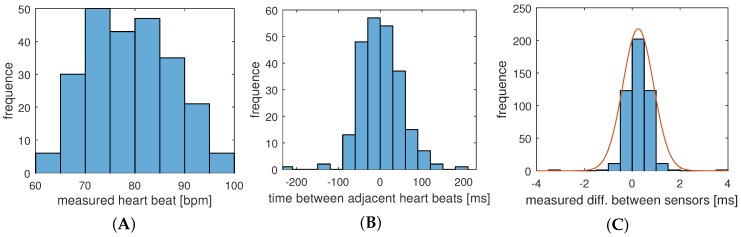
Distribution of (**A**) measured IPIs; (**B**) difference between two adjacent measurements; and (**C**) measurement errors between sensors.

A snippet of measured data for two nodes is shown in Table 3. We can see that both sensors deliver very similar Q, R, and S peaks, with only minor deviations. A histogram of the obtained IPIs, expressed as heart beats per minute is shown in Figure 11A. All measurements are between 60 and 100 bpm, without any uncharacteristic outliers. As underlying statistics for the authentication protocol, we further tracked how close the IPI values for adjacent heartbeats are, and how much timing error we observe between two nodes. The results for the two studies are shown in Figure 11B,C, respectively.

We see that adjacent heartbeats are related to each other, with a standard deviation of more than 60 ms. In contrast, our measurement error between the boards shows a standard deviation of $\sigma_{S}=0.74$ ms. Two orders of magnitude difference between measurement errors and natural uncertainty of the underlying phenomenon generally indicate the applicability of our system for a secure physiological authentication. The measurements were taken for a sampling rate of f=800 Hz. The uncertainty of the measurement error increases with lower sampling rates. For a sampling rate of f=666 Hz, we measured a standard deviation of $\sigma_{S}=6.7$ ms, and f=500 Hz resulted in $\sigma_{S}=110$ ms. We could not observe an improvement of the measurement precision for sampling rates above 800 Hz.

## 7. Protocol

In this section, we apply the results and measurements we gained for the system, presented in the previous section, to engineer and evaluate a secure and robust biometric authentication protocol. The major challenge is that different sensor nodes measure similar but not exactly the same values, so that a margin of error has to be accepted. In the authentication protocol, we exploit the fact, that the natural distribution of IPIs (see Figure 11B) is larger than the measured uncertainty of our system (Figure 11C). The hypothesis is that we can decide whether deviations for a set of sampling points from different sensors is caused by technical uncertainties or by a node that is not attached to the same body. Furthermore, our investigations address the question of how the number of samples and their resolution affect the quality and reliability of the authentication process.

### 7.1. Authentication Protocol

The authentication protocol we discuss in this section ensures that two nodes ($S_{1}$ and $S_{2}$) agree that they are attached to the same body, that is, they sense the same ECG data. We do not assume any specific network topology or hierarchy between $S_{1}$ and $S_{2}$. We further assume that $S_{1}$ and $S_{2}$ already agreed on a shared session key, which is used throughout the communication. That might be possible with Diffie–Hellman or other light-weight key agreement protocols [41].

**Figure 12:**
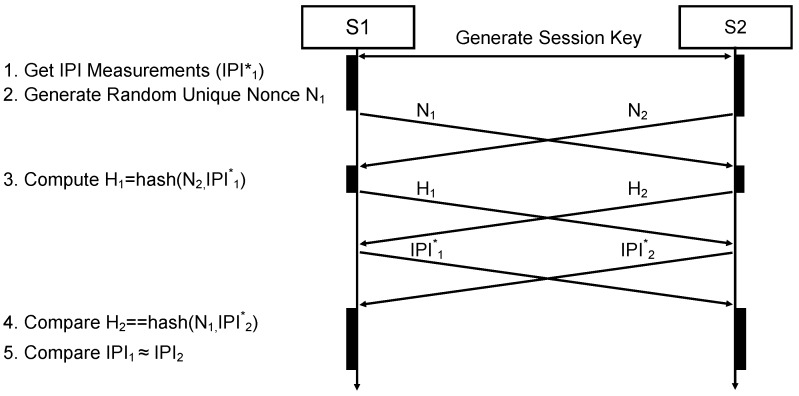
Message sequence chart for the biometric authentication protocol between Sensor nodes $S_{1}$ and $S_{2}$, with processing steps for $S_{1}$. The authentication is successful if the last two steps succeed.

A message sequence diagram of the physiological authentication protocol with its five steps is shown in Figure 12. The protocol starts with the session establishment and measurement of the IPIs. The key ideas of the protocol are:

1. The IPIs of the two peers are compared based on statistical properties in the $IPI_{1}\approx IPI_{2}$ operation in Step 5. The comparison operation (≈) compares the standard deviation of the differences between the two nodes and depends on a range of uncertainties and parameters that we discuss in the next subsection.
2. Each peer sends a hashed value of its measured IPIs before sending the actual IPI data (Step 3). Receiving the peer’s hash value (e.g., $H_{2}$) before sending the own IPIs ($IPI_{1}^{*}$), prevents the peer node from forging its IPI ($IPI_{2}^{*}$) after receiving the authentic data. If the received IPI does not match the received hash value (Step 4) , the authentication is invalid.
3. Possible replay attacks and simultaneous connection attempts are additionally hindered by enforcing the use of a unique random nonce $N_{i}$, which in our case is a 32 bit random integer number. The nonce is generated in Step 2, and has to be used by the peer for the hash generation (Step 3). Therefore, $H_{i}$ is the hash value of the concatenated received nonce and the array of measured IPI values.

A peer node is authenticated as a member of the BAN if the last two steps (4 and 5) succeed.

### 7.2. Parameters of the Authentication Protocol

Since participated sensor nodes might sense or process different IPI values, the comparison function $\approx (IPI_{1}^{*},IPI_{2}^{*})$ over the two IPI arrays of equal size has to accept a degree of uncertainty in the data values. Thereby, it is the goal to minimize the number of false positive authentications (FPA) and false negative authentications (FNA). 

**False negative authentications (NFA)** is the percentage of legitimate authentication attempts that are rejected due to a high level of measurement or processing errors.

**False positive authentications (NPA)** is the percentage of non-legitimate authentication attempts that are accepted by a node due to a high tolerance for measurement or processing errors. 

The comparison function $\approx (IPI_{1}^{*},IPI_{2}^{*})$ compares the statistical properties of the two IPI vectors. The function and its acceptance properties and its footprint can be tailored by three parameters: 

**Dynamic range (r)** measured in dB, expresses the resolution of the sampled IPI values. *r* is based on the distance Δ between two adjacent IPI values in ms. Since the underlying physical phenomenon is in a range of R=[0,2000] ms, we can compute the required number of bits per value by $b=log_{2}\frac{\left|R\right|}{\Delta }$, and the dynamic range is $r=20log_{10}\frac{\left|R\right|}{\Delta }$. Table 4 shows example values for Δ, *b*, and *r*. We assume that a lower dynamic range will reduce the number of false positives but increases the number of false negatives. 

It should be noted that changing the resolution also changes the statistical properties of the reference model. For instance the results in Figure 11 was taken with a resolution of r=0.5 ms and resulted in a standard deviation of $\sigma_{0.5ms}=1.28$, while a resolution of 2 ms results in $\sigma_{2ms}=0.45$.

**Number of samples (s)** defines the number of IPI data points we use for one authentication process. The number of samples influences the time for taking measurements, as well as the message size. Table 5 shows the message sizes for a range of configurations. From a quality perspective, we can expect that more samples compensate for outlier data points, and increase confidence in the positive or negative decision.

**Allowed deviation (d)** measured in number of standard deviations (*σ*), determines how much deviation is acceptable to distinguish legitimate measurements from data not originating from the same body. Therefore, *d* can be considered as similarity factor that decides whether a node is trusted or not. The value of *σ* is based on the actual measurements (see Figure 11) and the underlying dynamic range. Using the error factor $e=\frac{\sigma_{e}}{\sigma }$, while *σ* is the standard deviation of the expected measurement error (see Figure 11C), and $\sigma_{e}$ is the error distribution of the difference between $IPI_{1}$ and $IPI_{2}$:

(2) $$\sigma_{e}=\sqrt{\frac{1}{s}\sum_{i=1}^{s}\left(IPI_{1}\left[i\right]-IPI_{2}\left[i\right]\right)}$$

we define the comparison function as:

(3) $$\begin{array}{cc} \approx \left(IPI_{1}^{*},IPI_{2}^{*}\right)=\left\{\begin{array}{c} true\\ false \end{array}\right. & \begin{array}{c} \mathrm{iff}\;d\leq e\\ \mathrm{iff}\;d>e \end{array} \end{array}$$

It can be assumed that a larger *d* reduces the number of false negatives but increases the number of false positives.

The number samples (*s*) and the dynamic range (*r*) have a minor effect on the total computation time of the protocol. The protocol requires two hashing operations (of the nonce and the samples), and the computation of one standard deviation (of the differences between the measurements of the two peers). Depending on *s* and *r*, the total time for these operations varies between 130 μs and 160 μs when we apply MD5 as hash function, and between 250 μs and 290 μs when SHA256 is applied. These figures are negligible compared to the signal processing efforts reported in Section 6.3, and, therefore, do not affect the search for preferable parameter combinations.

### 7.3. Analysis

To identify superior combinations of parameters in this multi-dimensional space—and to validate the parameter assumptions expressed in the previous section—we executed a variety of tests with different variable and invariant parameters. All the experiments were executed in a Matlab environment using the authentication protocol proposed in Section 7.1. The expected reference error distribution and its standard deviation *σ* is provided by our practical sensor measurements (Figure 11). In the following subsection, we investigate the impact of the parameters on the FNA first, then study impact on the FPA, and conclude with the combined parameter selection and a sensitivity analysis.

#### 7.3.1. False Negatives Authentications (FNA)

**Figure 13:**
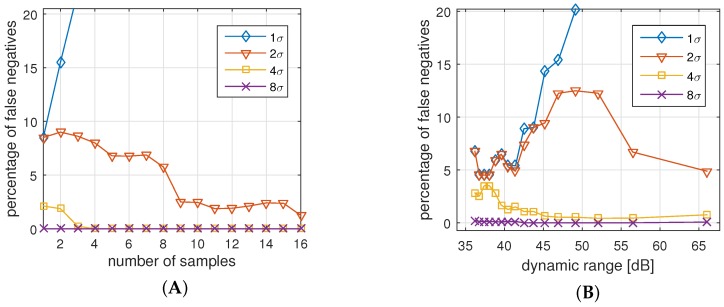
Rejected authentications (false negatives) for measured truthful authentication attempts, (**A**) for different number of samples; and (**B**) for different dynamic ranges.

Figure 13A,B show the rate of FNAs for different parameter settings. We applied the measured data for two sensors and applied different settings of sampling rate, dynamic range, and allowed deviation. The goal is to identify the sensitivity of the parameters to the legitimate authentication attempts.

Figure 13A shows the impact of the sample sizes for different allowed standard deviations, averaged for a invariant set of dynamic ranges. The results from this picture are:

- For all cases but d=1σ, an increased number of samples reduces the number of false negatives. The reason is that a higher number of samples help to reduce the effect of possible outliers.
- Allowing only a deviation of d=1σ is too strict for all settings. In fact, we see that higher number of samples increases the probability of a rejected authentication.
- With a deviation of d=4σ and more, and s=4 samples and more, we practically cannot identify any false negatives anymore, *i.e.*, all legitimate authentications are identified correctly.

Figure 13B shows the impact of the dynamic range for the same standard deviations. The results are similar to the previous results in a way that:

- Allowed deviation of d=1σ and 2σ results in too many errors regardless of the dynamic range.
- With a deviation of d=4σ and more, and a dynamic range of r=50 dB (8-bit) and more, we practically cannot identify any false negatives anymore, *i.e.*, authentication attempts are assessed correctly.

While the latter observation is most important for our parameter selection, it is worth noting that increasing the dynamic range does not generally reduce the FNR. One reason for this behavior could be that very high and very low resolutions might over-emphasize outliers, and therefore reject valid authentications.

#### 7.3.2. False Positive Authentication (FPA)

In this section, we investigate the impact of the parameters to non-legitimate authentication attempts. We assume the threat of similarity attacks [42] in which an adversary can exploit that values in an equivalence class are distinct but semantically similar. In other words, if an adversary can generate or guess similar IPI values, the presented protocol can be compromised. To generate non-legitimate IPIs, we assume a powerful attacker model in which an attacker knows the last legitimate sensor reading and is aware of the inter-IPI distribution (Figure 11B). Other models, such as simple guessing, or first order approximations did not result in observable successful authentications.

**Figure 14:**
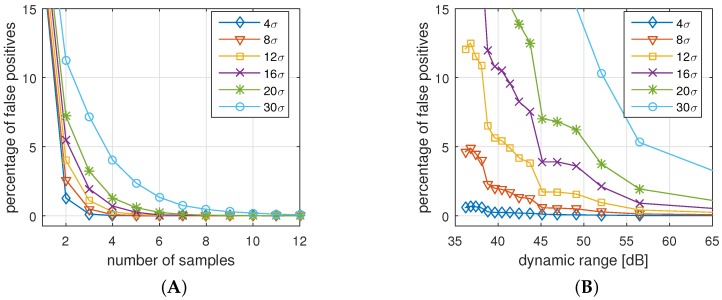
Accepted authentications (false positives) for forged authentication attempts, (**A**) for different number of samples; and (**B**) for different dynamic ranges.

Figure 14A,B show the FPA for different numbers of samples and dynamic ranges, respectively. The results in this case are:

- Increasing the number of samples or increasing the dynamic range always decreases the chance for a successful attack.
- Very high allowed deviations (d>10σ) improve the chances for a successful attack, while small allowed deviations (≤8σ) prohibit attacks.

### 7.4. Parameter Selection and Sensitivity Analysis

Based on the practical and analytical experimental results, the parameters of the comparison function ≈ can be set, so that the FPA and FNA rates are reduced, and the required resources (packet size) and authentication time (number of required samples) are considered. As a result, for our analysis of the data gathered with the presented BAN setup, we decided on a sample size of eight, with 8-bit sample encoding (48 dB) and an allowed deviation of 8σ. In our experiments, this setup led to 0% false positives and 0% false negatives. In fact, for the given setting, the allowed deviation *d* could be freely selected between 2 and 14 to obtain the same positive result.

To investigate the sensitivity of the parameter selection, in cases where the nodes process the data with a higher timing uncertainty than in our experiment, we ran tests with an increased noise level. Specifically, we studied the sensitivity of the accepted deviation *d* in environments with higher uncertainty. Possible reasons for higher deviations are less precise sensors and signal processing, timing-uncertainties in the embedded system, but also older or less healthy persons [18]. We were interested in the required thresholds of *d* to limit the FNR to 1%, and to limit the FPR to the levels of 1% and 5%.

**Figure 15:**
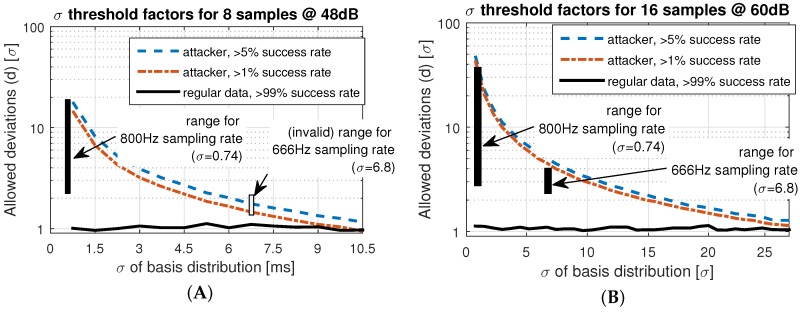
Impact of increased basis variance (e.g., more noise) on the allowed thresholds to limit false positives and false negatives, for (**A**) 8 samples at 48 dB, and (**B**) 16 samples at 60 dB.

The results are shown in Figure 15. Figure 15A shows the thresholds for our selected settings (eight samples, 48 dB). We see that the acceptable threshold for the FNR stays between 1σ and 2σ and is not affected by increased variation of the underlying measurements. The reason is that *σ* is a factor in the acceptance Equation (3), and, therefore, an increased noise level in the underlying distribution increases the acceptance deviation as well.

However, Figure 15A,B show that the thresholds for FPR decrease with an increased deviation in the reference error distribution. That effect is plausible, because if a high level of noise has to be tolerated, it is more challenging to differentiate between deviations due to attacks and natural noise.

Our default settings of the protocol, shown in Figure 15A, can tolerate measurement uncertainty up to σ=4 ms, if we require 1*σ* between the FPA and FNA thresholds. Higher noise levels would increase the FPAs, since attacks cannot be clearly distinguished from legitimate authentication attempts.

Larger uncertainty than σ=4 ms requires an adaptation of the system parameters. Figure 15B shows the thresholds for 16 samples and 60 dB (11 bit) dynamic range. The plot shows that, in this case, a 20 times higher basis deviation still provides sufficient space to differentiate between authentic and forged attempts. However, the cost for this improved confidence is the extended sample time (16 instead of 8 heart beats) and the larger packet sizes (160 instead of 64 byte).

To validate the simulation results, we applied practical measurements gathered with different sampling rates. The effective ranges for a 99% confidence of FPA and FNA are highlighted as the rectangles in Figure 15A,B. We only show the 800 Hz and 666 Hz sampling rates, because higher rates were not distinguishable from 800 Hz. Lower rates such as 500 Hz result in a standard deviation of σ>100 ms, which is impractical. The measurements show that the practical FNA threshold is about 1σ higher than estimated in the simulations, while the FPA threshold corresponds to the simulations. As a result, shown in Figure 15A, the data gathered at 666 Hz cannot be successfully distinguished, because with σ=6.8, the practical threshold to prevent attacks is lower than the threshold to identify legitimate attempts. With the extended setting (Figure 15B), we still have a valid range for *d* between 2.5*σ* and 4*σ*. The results show that protocol parameters with a preferable quality-to-resource trade-off can be found if the uncertainty of the implemented system are known.

## 8. Conclusions

The combination of real sensors, sophisticated protocols and system parameterization is never easy. This paper has shown how a physiological authentication protocol for body-area networks can be designed, implemented, and parametrized to work with the real-world uncertainties of low-cost body-area sensor nodes. The key for the presented authentication protocol is the statistical analysis of actual sensor measurements, which allows a designer to adapt system parameters in accordance with properties of real-world BAN deployments.

As a reproducible basis of our work, in the first part of this paper, we described how to design and implement the ECG sensor board and its processing system. In this part, it turned out that noise and signal quality had to be addressed in all design and processing steps, starting from analog pre-processing to the model-based validation step of the detected ECG peak signals. During the design, we further observed that model-based design flows are helpful in early design stages. However, to obtain a sufficient system performance, manual design efforts were required, in both the analog and the digital part of the system. The resulting sensor system is the first reported BAN sensor system to facilitate real-time tracking of ECG IPI data for inter-node authentication.

Practical measurements with the presented systems are the key for the design and the parametrization of the actual authentication protocol. With the statistical data of the practical behavior, we could define the allowable deviation margins so that honest authentications were permitted and false attempts could be prevented. We exploited the fact that the deviations of the underlying biometric property are higher than the the deviations of sensing and processing data to optimize and tailor the parameters of the authentication function and to reduce sensing and processing overhead. The recommended system configuration requires eight samples of 8-bit integer precision each, resulting in 100% correct authentications and an infeasible probability for a false positive authentication.

While the results of this first real-world biometric BAN system are promising, we also identified a range of possible future works. One future work is the extended validation of the protocol for more people in different situations and possible abnormal ECG properties. The goal would be a combination of biological sources of uncertainty as discussed in [18] and the technological aspects, discussed in our paper, to one framework to determine preferable system configurations to minimize the probability of errors. Another open research question is how can we make model-based design more effective, without the need of reimplementation of algorithms in a lower level implementation languages. The goal is the adaptation of the presented setup to an even smaller 8- or 16-bit computation platform. To reach that goal, extending the presented authentication scheme to generate secure session keys, instead of accepting pre-agreed session keys, could further improve the efficiency and applicability of the presented biometric authentication system.

## Figures and Tables

**Table 1:** Results for two sensors of the Matlab implementation on the PC.

IPI in (s)	1st IPI	2nd IPI	3rd IPI	4th IPI	5th IPI	6th IPI	7th IPI
Sensor 1 R	0.927	0.908	0.880	0.864	0.799	0.828	0.774
Sensor 2 R	0.927	0.908	0.880	0.865	0.799	0.828	0.774
Sensor 1 Q	0.926	0.909	0.880	0.865	0.798	0.829	0.774
Sensor 2 Q	0.926	0.908	0.879	0.865	0.799	0.828	0.775
Sensor 1 S	0.927	0.908	0.879	0.865	0.799	0.828	0.774
Sensor 2 S	0.927	0.908	0.880	0.865	0.799	0.827	0.775

**Table 2:** Results for two sensors of the Simulink implementation.

IPI in (s)	1st IPI	2nd IPI	3rd IPI	4th IPI	5th IPI	6th IPI	7th IPI
Sensor 1 R	0.927	0.908	0.880	0.864	1.299	0.828	0.774
Sensor 2 R	0.99	0.7737	0.73	0.908	0.8617	1.053	0.977
Sensor 1 Q	0.926	0.909	0.880	0.865	0.798	0.829	0.774
Sensor 2 Q	1.1723	0.828	1.0	1.086	0.9137	0.994	0.9873

**Table 3:** Results for two sensors of the C implementation on the BAN.

IPI in (s)	1st IPI	2nd IPI	3rd IPI	4th IPI	5th IPI	6th IPI
Sensor 1 Q	0.965	0.967	0.901	0.964	0.984	0.913
Sensor 2 Q	0.965	0.969	0.905	0.960	0.986	0.910
Sensor 1 R	0.964	0.969	0.904	0.960	0.986	0.910
Sensor 2 R	0.965	0.969	0.905	0.960	0.986	0.910
Sensor 1 S	0.964	0.968	0.901	0.963	0.985	0.913
Sensor 2 S	0.965	0.969	0.904	0.962	0.985	0.910

**Table 4:** Quantization per sample.

Distance Δ (ms)	Req. Bits *b*	Dyn. Range *r* (dB)
1	11	66
2	10	60
4	9	53
8	8	47
20	7	40

**Table 5:** Example signature sizes.

Distance Δ	Samples			
[ms]	2	4	8	16
2	20	40	80	160
4	18	36	72	144
8	16	32	64	128
20	14	28	56	112
